# Spatio-Temporal Joint Optimization-Based Trajectory Planning Method for Autonomous Vehicles in Complex Urban Environments

**DOI:** 10.3390/s24144685

**Published:** 2024-07-19

**Authors:** Jianhua Guo, Zhihao Xie, Ming Liu, Zhiyuan Dai, Yu Jiang, Jinqiu Guo, Dong Xie

**Affiliations:** 1 State Key Laboratory of Automotive Simulation and Control, Jilin University, No. 5988, Renmin Street, Nanguan District, Changchun 130022, China; jiezh22@mails.jlu.edu.cn (Z.X.); daizy22@mails.jlu.edu.cn (Z.D.); 15615630556@163.com (Y.J.); jqguo23@mails.jlu.edu.cn (J.G.); xiedongjlu@163.com (D.X.); 2 School of Automotive Studies, Tongji University, Shanghai 201804, China; 2110215@tongji.edu.cn

**Keywords:** autonomous vehicles navigation, motion planning, collision avoidance

## Abstract

Providing safe, smooth, and efficient trajectories for autonomous vehicles has long been a question of great interest in the field of autopiloting. In dynamic and ever-changing urban environments, safe and efficient trajectory planning is fundamental to achieving autonomous driving. Nevertheless, the complexity of environments with multiple constraints poses challenges for trajectory planning. It is possible that behavior planners may not successfully obtain collision-free trajectories in complex urban environments. Herein, this paper introduces spatio–temporal joint optimization-based trajectory planning (SJOTP) with multi-constraints for complex urban environments. The behavior planner generates initial trajectory clusters based on the current state of the vehicle, and a topology-guided hybrid A* algorithm applied to an inflated map is utilized to address the risk of collisions between the initial trajectories and static obstacles. Taking into consideration obstacles, road surface adhesion coefficients, and vehicle dynamics constraints, multi-constraint multi-objective coordinated trajectory planning is conducted, using both differential-flatness vehicle models and point-mass vehicle models. Taking into consideration longitudinal and lateral coupling in trajectory optimization, a spatio–temporal joint optimization solver is used to obtain the optimal trajectory. The simulation verification was conducted on a multi-agent simulation platform. The results demonstrate that this methodology can obtain optimal trajectories safely and efficiently in complex urban environments.

## 1. Introduction

With the advancement of intelligent technologies, autonomous vehicles (AVs) are increasingly being deployed in urban environments. An autonomous driving system comprises perception, localization, planning, and control [1]. As a fundamental aspect of autonomous driving technology, trajectory planning significantly enhances traffic safety, improving traffic efficiency, reducing congestion and mitigating environmental pollution [2,3,4]. Significant progress has been made in trajectory planning for autonomous vehicles in urban environments. However, the task of generating safe and efficient trajectories for autonomous vehicles in urban areas is significantly challenging, due to multiple factors. These include the prominent role that vulnerable road users (VRUs) play in complex urban scenarios, which increases the risk of collisions [5], as well as the high-density traffic flows, diverse vehicle types, and irregular obstacles that characterize these environments. In recent years, numerous researchers have focused on the study of trajectory planning for autonomous vehicles in urban settings.

Trajectory planning can be divided into the pipeline planning method and the end-to-end planning method [1]. However, the complexity of urban environments, characterized by numerous dynamic obstacles, complicates data collection. Additionally, the low interpretability of end-to-end planning methods further limits their practicality in real-world applications [1,6,7]. Conversely, a significant advantage of the pipeline planning method is interpretability, which aids in identifying malfunctioning modules when errors occur [1]. For the classic pipeline planning method, sampling-based methods and numerical optimization methods are widely used in urban scenarios.

Typical sampling-based methods, such as state lattice and probabilistic planners, generate feasible trajectories from an initial point to a target point by sampling the vehicle’s state. Lattice-based planners [8,9] discretize the continuous state space into a lattice graph by sampling trajectory parameters, including running time (t), longitudinal displacement (s), and lateral displacement (d) along a reference line. Subsequently, the control system selects and executes the trajectory that receives the highest score based on specific evaluation criteria from among the candidates. The rapidly exploring random tree (RRT) [10,11,12,13] is a typical probabilistic planner that aims to facilitate path planning by randomly sampling within the state space and extending towards these samples to form a trajectory. Cheng et al. [14] modeled path planning as probabilistic inference using Gaussian processes (GPs), applying an s–t graph search to generate and iteratively refine velocity profiles along paths, ensuring kinematic feasibility. The quality of the trajectories produced by the majority of these methods depends on the number of candidate trajectories [15]. However, dense sampling is time-consuming, and sparse sampling often results in suboptimal solutions. As a result, this method struggles to generate optimal trajectories in complex scenarios.

Optimization-based algorithms can enhance the initial trajectories generated by sampling-based planning algorithms [16,17,18,19]. By integrating constraints related to smoothness, safety, and other factors, these algorithms aim to find the optimal solution for the objective function within the specified constraints. The improved trajectories align more closely with the real-world requirements of driving. In urban environments, the Frenét frame is commonly employed, due to its effective representation of lane geometric constraints [14,20,21,22,23]. Among the employed methodologies, two particular methods are notable: the EM planner [20] and the spatial–temporal safe corridor (SSC) [21]. The EM planner was developed to tackle the intricate three-dimensional (SLT) optimization problem characterized by the longitudinal (s), lateral (l), and temporal (t) dimensions. This is achieved by dividing it into two separate two-dimensional optimization challenges: S–T (longitudinal–temporal) and S–L (longitudinal–lateral). Path and speed planning are facilitated through the use of dynamic programming and quadratic programming, respectively. It is important to note that the convergence speed of this method may be relatively slow in environments populated with numerous dynamic obstacles. On the other hand, the SSC method introduces a spatial–temporal safe corridor to ensure dynamic safety based on the Frenét frame. This method optimizes trajectories within a defined semantic corridor, thereby generating physically feasible and collision-free trajectories. However, the fixed time resolution constrains further enhancements in efficiency. Model predictive control (MPC) methods typically formulate the trajectory planning problem as an optimal control problem (OCP), subsequently discretizing it into a nonlinear programming (NLP) problem [24]. These methods, as discussed in Refs. [25,26,27,28], concentrate on optimizing discrete states and control inputs while considering road boundary constraints and the uncertainties associated with other traffic participants. MPC-based methods include some typical methods as well. Zhang et al. [29] proposed the optimization-based collision avoidance (OBCA) algorithm, which precisely reformulates non-differentiable collision constraints into smooth and differentiable constraints. In a subsequent study, Zhang et al. [30] combined OBCA with MPC to introduce the hierarchical OBCA (H-OBCA) algorithm, which has achieved notable results. Dixit et al. [28] employed a robust model predictive control method integrated with potential field functions to facilitate high-speed autonomous overtaking, ensuring safety and kinematic feasibility through both lateral and longitudinal vehicle maneuvers, validated via high-fidelity simulations. Although the optimization-based algorithms mentioned above generally enhance the quality of trajectory, their impact on improving traffic efficiency is often marginal. The number of trajectory constraint points should be significantly increased in complex urban environments, which escalates the complexity of the optimization process, thereby substantially compromising the real-time capability of trajectory planning [15].

Joint optimization of time and space enables the full utilization of the solution space to generate trajectories that enhance traffic efficiency while ensuring safety. Han et al. [15] utilized the differential flatness property of the vehicle system to connect the trajectory with the vehicle’s kinematic parameters, thereby constraining those parameters. Static-obstacle-avoidance constraints were imposed by constructing driving corridors. The signed distance represented the distance between the ego vehicle and other vehicles, and dynamic obstacle avoidance was achieved by limiting the lower bound of the signed distance. The banded matrix was employed to ensure the continuity of position, velocity, and acceleration of the trajectory at waypoints, and to enforce constraints at the start and end points. Finally, the constrained optimization problem was converted into an unconstrained one, and it was solved using the quasi-Newton method [31]. The final result not only fulfills safety requirements but also ensures quality, enhancing traffic efficiency while meeting real-time demands. In high-speed scenarios, the road surface condition is critical for planning and control, as it determines the upper limit of the vehicle’s force, which influences the kinematic constraints on the maximum longitudinal and lateral accelerations. However, this method lacks discussion on the road surface. Meanwhile, in complex urban environments, while the driving corridor can effectively address static obstacle avoidance and optimize trajectories within it to achieve favorable outcomes, there is a possibility that the driving corridor may fail to generate, due to the initial trajectory generated by the behavior planner, and that it will not be collision-free.

**Figure 1:**
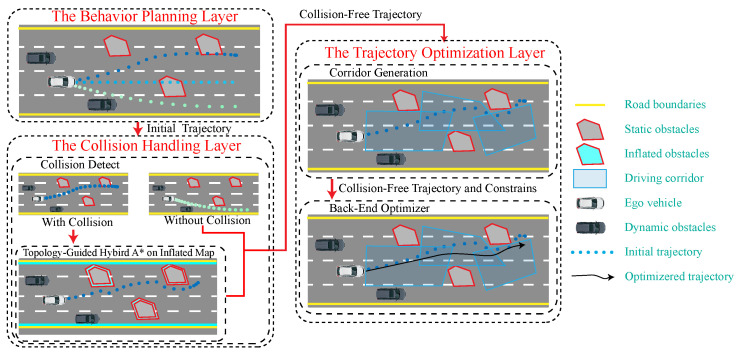
The trajectory planning framework.

Given the above challenges, this paper proposes a spatio–temporal joint optimization-based trajectory planning (SJOTP) method for autonomous vehicles for complex urban environments, as illustrated in Figure 1. The framework consists of three layers: the behavior planning layer, the collision handling layer, and the trajectory optimization layer. The behavior planner generates an initial trajectory based on the relationship between the current state of the vehicle and the target location; this initial trajectory undergoes collision detection with static obstacles. In cases where a collision occurs, a topology-guided hybrid A* algorithm on an inflated map is utilized to create a collision-free trajectory, which replaces the segment of the initial trajectory involved in the collision. Based on the collision-free trajectory and the vehicle driving direction, trajectory points are expanded to form a driving corridor, ensuring that the entire trajectory remains within a confined space defined by free convex polygons. Considering vehicle dynamics, safety corridors, dynamic obstacles, and other derived constraints, this method employs spatio–temporal joint optimization to coordinate multiple objectives, including safety, efficiency, and comfort, thereby generating smooth, safe, and efficient trajectories. The contributions of this paper can be summarized as follows:

- A topology-guided hybrid A* strategy is implemented on an inflated map, to overcome challenges associated with generating driving corridors when the initial trajectory is not collision-free.
- Considering multi-constraint limitations, including the road surface adhesion coefficient, multi-objective coordination for trajectory optimization is carried out, achieving the generation of safe, high-quality trajectories under various road surface adhesion coefficients.
- Considering the strong coupling relationship between the longitudinal and lateral movements of the vehicle, a spatio–temporal joint optimization solver is used to simultaneously optimize the longitudinal and lateral initial values of the trajectory.

The remaining content is organized as follows: Section 2 describes the problem, formulating the trajectory optimization and constraints. The main framework and specific methods are introduced in Section 3. Section 4 presents simulations and comparative experiments. The article concludes in Section 5.

## 2. Problem Description

### 2.1. Trajectory Planning Scenarios

**Figure 2:**
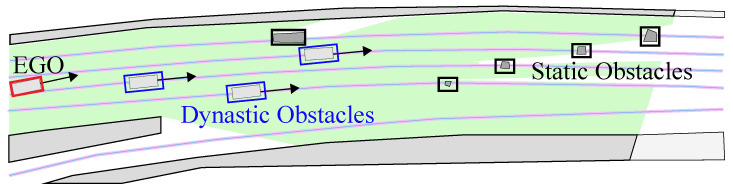
Trajectory planning scenarios.

This study focuses on scenarios within complex urban road environments. As illustrated in Figure 2, the presence of static obstacles, dynamic obstacles, and road boundaries in the environment poses significant challenges for the trajectory planning of autonomous vehicles. The goal of trajectory planning is to find a smooth, safe, and efficient trajectory. Smoothness is quantified by curvature. Efficiency is evaluated by the vehicle’s traversal time through complex scenarios or its average speed. To ensure safety, there must be no collision with static or dynamic obstacles.

### 2.2. Differentially Flat Vehicle Model

**Figure 3:**
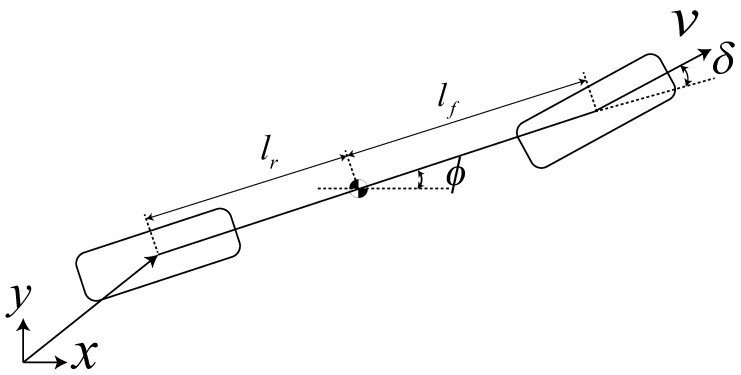
The kinematic bicycle model.

If flat outputs and their higher-order derivatives can smoothly describe a system’s state and if inputs can be identified then the system is considered differentially flat [32]. Vehicles are typically differentially flat systems. A simplified kinematic bicycle model is used to describe a four-wheel vehicle, assuming the model is not slipping, with steerable front wheels and fixed rear wheels. The model can be described as Figure 3. The state vector of the system is selected as

(1) $$x={\left(p_{x},p_{y},\theta ,v,a_{t},a_{n},\phi ,\kappa \right)}^{T}$$

where $p={\left(p_{x},p_{y}\right)}^{T}$ is position at the center of the rear axis, *v* is longitudinal velocity, ϕ is heading angle, δ is the steering angle of the front wheels and κ is the curvature, $a_{t}$ represents the longitude acceleration, and $a_{n}$ represents the lateral acceleration. We choose $\sigma =\left(\sigma_{x},\sigma_{y}\right)$ as the flat output, with a physical meaning that σ=p. Based on the differential flatness properties of the vehicle system, other variables related to the vehicle kinematics can be expressed as

(2) $$\left\{\begin{array}{c} v=\eta \sqrt{\sigma_{x}^{2}+\sigma_{y}^{2}}\\ \phi =\arctan2(\eta {\dot{\sigma }}_{y},\eta {\dot{\sigma }}_{x})\\ a_{t}=\eta \left({\dot{\sigma }}_{x}{\ddot{\sigma }}_{x}+{\dot{\sigma }}_{y}{\ddot{\sigma }}_{y}\right)/\sqrt{{\dot{\sigma }}_{x}^{2}+{\ddot{\sigma }}_{y}^{2}}\\ a_{n}=\eta \left({\dot{\sigma }}_{x}{\ddot{\sigma }}_{y}-{\dot{\sigma }}_{y}{\ddot{\sigma }}_{x}\right)/\sqrt{{\dot{\sigma }}_{x}^{2}+{\dot{\sigma }}_{y}^{2}},\\ \theta =\arctan(\eta \left({\dot{\sigma }}_{x}{\ddot{\sigma }}_{y}-{\dot{\sigma }}_{y}{\ddot{\sigma }}_{x}\right)L/{\left({\dot{\sigma }}_{x}^{2}+{\dot{\sigma }}_{y}^{2}\right)}^{\frac{3}{2}})\\ \kappa =\eta \left({\dot{\sigma }}_{x}{\ddot{\sigma }}_{y}-\sigma_{y}{\ddot{\sigma }}_{x}\right)/{\left({\dot{\sigma }}_{x}^{2}+{\dot{\sigma }}_{y}^{2}\right)}^{\frac{3}{2}} \end{array}\right.$$

In urban road scenarios, the variable η∈{1} means that only forward movement of the vehicle is allowed, while in unstructured roads, such as parking areas, the variable η∈{−1,1} means that the vehicle is allowed to forward and reverse.

### 2.3. Trajectory Planning Formulation

To improve the accuracy of trajectory tracking and ensure the comfort of passengers, the trajectory planning of autonomous vehicles should meet multi-dimensional constraints. The planned trajectory should satisfy the continuity criteria, encompassing the continuity of position, velocity, and curvature. Additionally, the trajectory should take into account the vehicle’s kinematic constraints. Most importantly, trajectory planning must ensure safety, which means no collision with static and dynamic obstacles. Trajectory planning should be transformed into an optimization problem aimed at maximizing the smoothness and efficiency of the trajectory. This optimization must consider several key constraints, including trajectory continuity, vehicle kinematics, and obstacle avoidance.

Due to the jerk-optimal nature of the quintic polynomial, M-piece quintic polynomials are used to connect waypoints $P=\left(P_{1},\cdots ,P_{M-1}\right)\in R^{2\times (M-1)}$, the starting point, and the end point. The *i*-th segment trajectory $\sigma_{i}$ is written as follows:

(3) $$\begin{array}{ccc} \; & \sigma_{i}\left(t\right)=a_{i}^{T}\alpha_{i}\left(t\right) & \\ \; & \alpha_{i}\left(t\right)={\left(1,\delta t_{i},\delta t_{i}^{2},\delta t_{i}^{3},\delta t_{i}^{4},\delta t_{i}^{5}\right)}^{T} & \\ \; & \forall i\in \{1,2,3,\ldots ,M\} \end{array}$$

where $\delta t_{i}$ is the *i*-th piece time interval, $\alpha_{i}\left(t\right)$ is a natural basis, $a={\left(a_{1}^{T},\cdots ,a_{M}^{T}\right)}^{T}\in R^{6M\times 2}$ is the coefficient matrix, $\sigma_{i}$ is the *i*-th piece position at the center of the rear axis, and $T_{a}=\sum_{i=1}^{M}\delta t_{i}$ is the duration of the whole trajectory. With constraints for safety and dynamic feasibility, the problem of minimizing control effort with time regularization can be formulated as a nonlinear constrained optimization problem:

(4) $$\begin{array}{c} \min_{c,T}J\left(c,T\right)=\int_{0}^{T_{a}}u{\left(t\right)}^{T}Wu\left(t\right)dt+w_{T}T_{a}\\ s.t.u\left(t\right)=\sigma^{\left[3\right]}\left(t\right),\forall t\in \left[0,T_{a}\right],\\ \sigma_{0}^{\left[j\right]}\left(0\right)={\bar{\sigma }}_{0},\sigma_{M}^{\left[j\right]}\left(\delta t_{M}\right)={\bar{\sigma }}_{f},0⩽j<3\\ \sigma_{i}^{\left[j\right]}\left(\delta t_{i}\right)=\sigma_{i+1}^{\left[j\right]}\left(0\right),1⩽i<M,0⩽j<3\\ \delta t_{i}>0,1⩽i<M\\ C_{d}\left(\sigma \left(t\right),\ldots ,\sigma^{\left(s\right)}\left(t\right),t\right)⩽0,\forall d\in D,\forall t\in \left[0,T_{a}\right] \end{array}$$

where $W\in R^{2\times 2}$ is a diagonal matrix to penalize control efforts, and where ${\bar{\sigma }}_{0},{\bar{\sigma }}_{f}\in R^{2\times 3}$ refers to the position, velocity, and acceleration of the trajectory at the starting and end points. The second term $w_{T}T_{a}$ in the objective function is the time regularization term to restrict total duration $T_{a}$, with a weight $w_{T}\in R^{+}$. The constraint function at *d* is defined as $C_{d}$, the set $D=\left\{d:d=v,a_{t},a_{n},\kappa ,\zeta_{s},\zeta_{y}\right\}$ includes dynamic feasibility $v,a_{t},a_{n},\kappa$, and static and dynamic obstacle avoidance $\zeta_{s},\zeta_{y}$. However, dynamic feasibility constraints, such as road surface conditions, significantly impact trajectory planning but are often overlooked in current research. Furthermore, in obstacle avoidance, the potential for failures in generating driving corridors must be considered. These are crucial issues in vehicle trajectory planning that need to be addressed.

The constraints in the set D are essentially inequalities, and the inequality constraints in Equation (4) can be converted into a penalty term $S_{\Sigma }\left(c,T\right)$, resulting in an unconstrained nonlinear optimization problem [33]:

(5) $$\min_{c,T}J\left(c,T\right)=\int_{0}^{T_{a}}u{\left(t\right)}^{T}Wu\left(t\right)dt+w_{T}T_{a}+S_{\Sigma }\left(c,T\right)$$

The trajectory is discretized into multiple constraint points according to a fixed number *K*. To satisfy the inequality constraints, each constraint point is penalized, and the penalty expression is

(6) $$S_{\Sigma }=\sum_{d\in D}w_{d}\sum_{i=1}^{M}\sum_{j=1}^{K}P_{d,i,j}\left(c_{i},T\right),$$

(7) $$P_{d,i,j}\left(c_{i},T\right)=\frac{\delta t_{i}}{K}L_{1}\left(G_{d,i,j}\right),$$

where $w_{d}$ is the penalty weight and L1(•) is the L1-norm relaxation function [34].

## 3. SJOTP-Based Planning

### 3.1. The Behavior Planning Layer

An important module of autonomous vehicles is the behavior planner, which primarily serves as a decision-making module to ensure safe driving and effective obstacle avoidance. The behavior planner is integrated with a trajectory planner to enhance decision-making capabilities for tasks such as lane selection. For the behavior planning layer, various methodologies are available. In our proposed framework, we have chosen to implement a high-level decision-making approach through a multi-agent simulation, as detailed in [35]. This method effectively addresses the uncertainties inherent in real-time driving environments and ensures robust performance of the system in real-time conditions.

### 3.2. The Collision Handling Layer

In complex urban scenarios, a prerequisite and key to generating driving corridors is obtaining a collision-free trajectory. However, the initial trajectory from the behavior planner may collide with static obstacles on structured roads, leading to the failure of driving corridor generation. To address this issue, we employ a hybrid A* algorithm to generate a collision-free trajectory that effectively replaces the segment of the initial trajectory that collides with obstacles. However, it is essential to take into account the homotopy of the trajectories generated by the hybrid A* algorithm in two consecutive planning sessions. For a vehicle with nonholonomic dynamics navigating structured roads at high speeds, adjusting swiftly to changes in trajectory topology presents a considerable challenge. Non-homotopic trajectories elevate the risk of collisions with static obstacles, particularly when the vehicle is near them.

Inspired by [34], when the initial trajectory derived from the behavior planner of this cycle encounters a collision, we utilize collision detection to ascertain appropriate start and end points for the hybrid A* algorithm, which remains applicable even with multiple obstacle collisions. Then, we check whether the initial trajectory from the previous cycle encounters obstacles. Without collisions, the trajectory is directly computed, using the hybrid A* algorithm with the start and end points as inputs. In the case of collisions, the collision-free trajectory $\tau_{0}\left(s\right)$ from the previous hybrid A* search is utilized for generating the collision-free trajectory of this cycle τ(s).

**Figure 4:**
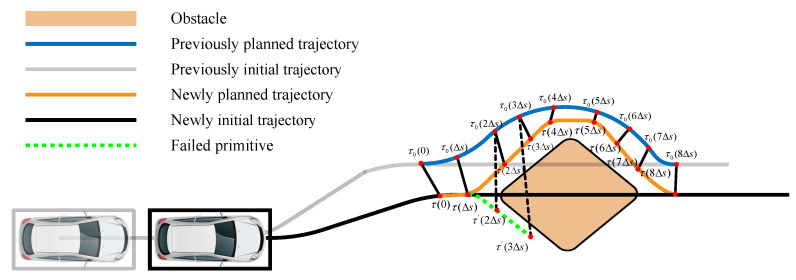
Topology-guided hybrid A* for urban scenarios.

**Figure 5:**
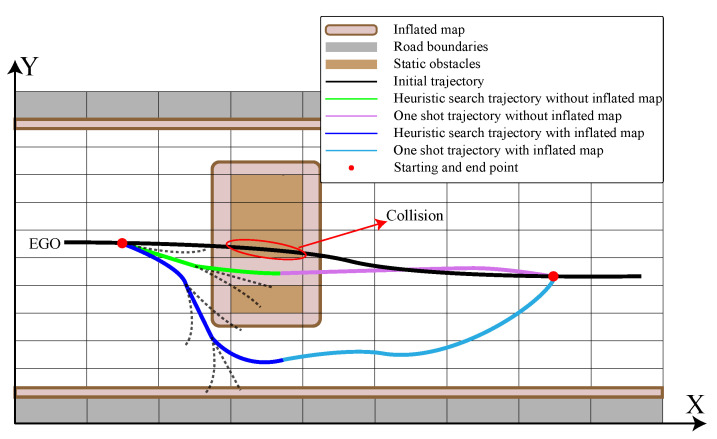
Application of Hybrid A* algorithm on an inflated map.

If we assume that each expanded primitive shares a fixed arc length Δs and that the position of the *k*-th primitive starting from the starting point is τ(kΔs) then the point $\tau_{0}\left(s_{0}\right)$ on the collision-free trajectory from the previous cycle, closest to the starting point of the current cycle, can be found. Then, we iteratively check if line $\tau \left(k\Delta s\right)-\tau_{0}\left(s_{0}+k\Delta s\right)$ is collision-free and if $|\tau \left(k\Delta s\right)-\tau_{0}\left(s_{0}+k\Delta s\right)|{|}_{2}\leq D_{th}$, where $D_{th}$ is the threshold parameter. If either of the above requirements is not satisfied, the newly searched primitive will not be accessed anymore. The topology-guided hybrid A* search process for urban scenarios is illustrated in Figure 4:

In addition to the requirement for the trajectory to satisfy homotopy, the distance between the obstacles on either side of the expanded node should exceed the vehicle’s width. Otherwise, even if a driving corridor can be generated, the vehicle cannot pass through safely. The solution is implemented using an inflated map, where obstacles and road boundaries are expanded by a predefined threshold value, denoted as $w_{th}=\frac{w}{2}+d_{safe}$, where *w* represents the width of the ego vehicle and $d_{safe}$ is the parameter to increase the safety margin. As long as the hybrid A* algorithm can find a trajectory to the end point, the vehicle can pass through. As illustrated in Figure 5, before map inflation, the green line represents a trajectory that should be excluded from the search but is mistakenly included. After map inflation, the desired trajectory indicated by the blue line is successfully identified.

### 3.3. The Trajectory Optimization Layer

In this section, we delve into the constraints associated with trajectory planning, including dynamic feasibility and obstacle avoidance. As described in Section 2, both dynamic feasibility and obstacle avoidance are essentially inequality constraints. By penalizing trajectories that violate these constraints, the constrained optimization problem can be transformed into an unconstrained one. The spatio–temporal joint trajectory optimization [15] applied in this chapter also adopts this method. Requiring only a single differentiation of the given flat dynamics and with the available gradients, the optimization objective can be solved using the L-BFGS algorithm [31]. This space-and-time joint trajectory optimization ensures that the generated trajectories not only adhere to dynamic and safety constraints but also optimize performance metrics, resulting in smooth and efficient paths.

#### 3.3.1. Dynamic Feasibility

1. Longitude velocity limit: To comply with traffic rules, accounting for user settings and traffic conditions, autonomous vehicles need to limit the maximum longitudinal velocity within a reasonable range:
  (8) $$C_{v}\left(\dot{\sigma }\right)={\dot{\sigma }}^{T}\dot{\sigma }-v_{m}^{2}$$
  where $v_{m}$ is the maximum longitudinal speed.
2. Acceleration limit: The frictional limit between the tire and the road surface is greatly influenced by the road conditions. During the driving process of the autonomous vehicle, it is necessary to consider the limitation of the road surface adhesion coefficient. With a low road surface adhesion coefficient, excessive longitudinal acceleration can provoke tire slip, and excessive lateral acceleration can provoke the vehicle to sideslip. Therefore, it is necessary to appropriately limit the maximum longitudinal and lateral acceleration of autonomous vehicles. The acceleration constraint is translated as follows:
  (9) $$C_{a_{t}}\left(\dot{\sigma },\ddot{\sigma }\right)=\frac{{\left({\ddot{\sigma }}^{T}\dot{\sigma }\right)}^{2}}{{\dot{\sigma }}^{T}\dot{\sigma }}-a_{tm}^{2},$$
  (10) $$C_{a_{n}}\left(\dot{\sigma },\ddot{\sigma }\right)=\frac{{\left({\ddot{\sigma }}^{T}B\dot{\sigma }\right)}^{2}}{{\dot{\sigma }}^{T}\dot{\sigma }}-a_{nm}^{2},$$
  where $B=\left[\begin{array}{cc} 0 & -1\\ 1 & 0 \end{array}\right]$ is an auxiliary antisymmetric matrix.
  The point mass vehicle kinematic model simplifies the vehicle’s dimensional information and ignores the influence of load transfer due to lateral and longitudinal acceleration. This model describes the vehicle’s motion as a point with mass, thereby effectively reducing the computational load for trajectory planning. Consequently, it is necessary to ensure that the total forces of the vehicle remain within the limits provided by the road surface, satisfying the friction circle constraint:
  (11) $$\begin{array}{c} ma_{t}=F_{x}\\ ma_{n}=F_{y}\\ F_{a}=\sqrt{F_{x}^{2}+F_{y}^{2}}\leq k\mu F_{z}=k\mu mg \end{array}$$
  where $F_{a}$ is the total force acting on the vehicle, $F_{x}$ is the longitudinal force, $F_{y}$ is the lateral force, μ is the road surface adhesion coefficient, and k≤1 is a proportionality coefficient aimed at limiting friction saturation. In accordance with the theory of the friction circle, it is imperative to guarantee that the total force exerted does not surpass the maximum frictional force. The friction circle constraint function, utilizing flat outputs, is defined as follows:
  (12) $$C_{F_{a}}\left(\dot{\sigma },\ddot{\sigma }\right)=\frac{{\left({\ddot{\sigma }}^{T}\dot{\sigma }\right)}^{2}}{{\dot{\sigma }}^{T}\dot{\sigma }}+\frac{{\left({\ddot{\sigma }}^{T}B\dot{\sigma }\right)}^{2}}{{\dot{\sigma }}^{T}\dot{\sigma }}-{\left(kug\right)}^{2},$$
  According to the chain rule, the gradients of $C_{F_{a}}\left(\dot{\sigma },\ddot{\sigma }\right)$ are derived as
  (13) $$\frac{\partial C_{F_{a}}}{\partial \dot{\sigma }}=2\frac{{\ddot{\sigma }}^{T}\dot{\sigma }}{\left|\right|\dot{\sigma }{\left|\right|}_{2}^{2}}\ddot{\sigma }-2(\frac{{\ddot{\sigma }}^{T}\dot{\sigma }}{\left|\right|\dot{\sigma }{\left|\right|}_{2}^{2}}{)}^{2}\dot{\sigma }+2\frac{{\ddot{\sigma }}^{T}B\dot{\sigma }}{\left|\right|\dot{\sigma }{\left|\right|}_{2}^{2}}B^{T}\ddot{\sigma }-2(\frac{{\ddot{\sigma }}^{T}B\dot{\sigma }}{\left|\right|\dot{\sigma }{\left|\right|}_{2}^{2}}{)}^{2}\dot{\sigma }$$
  (14) $$\frac{\partial C_{F_{a}}}{\partial \ddot{\sigma }}=2\frac{{\ddot{\sigma }}^{T}\dot{\sigma }}{\left|\right|\dot{\sigma }{\left|\right|}_{2}^{2}}\dot{\sigma }+2\frac{{\ddot{\sigma }}^{T}B\dot{\sigma }}{\left|\right|\dot{\sigma }{\left|\right|}_{2}^{2}}B\dot{\sigma }$$
3. Curvature limit: For high-speed autonomous vehicles, limiting curvature is crucial, due to the significant impact of curvature on vehicle stability and safety. The curvature constraint function is defined as follows:
  (15) $$C_{\kappa_{l}}\left(\dot{\sigma },\ddot{\sigma }\right)=\frac{{\ddot{\sigma }}^{T}B\dot{\sigma }}{\left|\right|\dot{\sigma }{\left|\right|}_{2}^{3}}-\kappa_{m},$$
  (16) $$C_{\kappa_{r}}\left(\dot{\sigma },\ddot{\sigma }\right)=-\kappa_{m}-\frac{{\ddot{\sigma }}^{T}B\dot{\sigma }}{\left|\right|\dot{\sigma }{\left|\right|}_{2}^{3}}.$$

#### 3.3.2. Obstacle Avoidance

In structured road environments, autonomous vehicles need to frequently re-plan, to avoid collisions with static and dynamic obstacles. The key to static obstacle avoidance is decomposing the semantic environment, to extract the free space for vehicle maneuvering. Constructing a driving corridor is a common and effective method for autonomous vehicles to avoid static collisions. The driving corridor consists of a series of convex polygons and can be constructed by expanding collision-free trajectory points in predefined directions on a semantic map [36] or directly from the point cloud [37].

**Figure 6:**
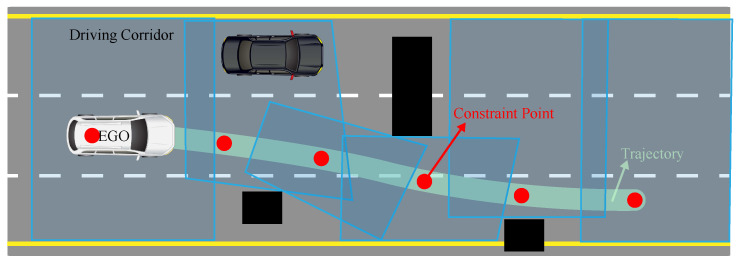
Driving corridors generation on structured roads.

To ensure collision avoidance with static obstacles, the vehicle’s outline is maintained entirely within the driving corridor, the polygon denoted as *E*; the vertices of the vehicle are denoted as *V*:

(17) $$V=\left\{v_{i}\in R^{2}:v_{i}=\sigma +Rl_{i},i=1,2,...,n_{i}\right\},$$

where *R* is the rotation matrix from the body to the world frame and $R=\frac{\eta }{\left|\right|\dot{\sigma }|{|}_{2}}\left(\dot{\sigma },B\dot{\sigma }\right)$, $n_{i}$ is the number of vertices, and $l_{i}$ is the coordinate of the *i*-th vertex in the body frame. The H-representation [38] of each convex polygon $P^{H}$ in the driving corridor is obtained:

(18) $$\begin{array}{c} P^{H}=\left\{q\in R^{2}:Aq\leq b\right\},\\ A={\left(A_{1},\ldots ,A_{p},\ldots ,A_{n_{p}}\right)}^{T}\in R^{n_{p}\times 2},\\ b={\left(b_{1},...,b_{p},...,b_{n_{p}}\right)}^{T}\in R^{n_{p}}, \end{array}$$

where $n_{p}$ is the number of hyperplanes, and $A_{p}\in R^{2}$ and $b_{p}\in R$ represent the parameters describing a hyperplane. Due to the convexity of the driving corridor, the sufficient and necessary condition to ensure that the vehicle’s outline remains within the driving corridor is that the vertices of the vehicle’s outline are within the driving corridor. The generation of driving corridors in complex urban scenarios is illustrated in Figure 6.

To ensure that the planned trajectory is not overly conservative and to reduce computational complexity when facing dynamic obstacles, unlike other papers [14,36] that model the ego vehicle and other agents as a union of circles, both the ego vehicle and other agents are modeled as convex polygons. Convex set collision-avoidance methods typically penalize the signed distance between two sets. The signed distance is determined by both the distance and the penetration depth. The distance is defined as the minimum translation T required to separate the sets:

(19) $$\mathrm{dist}\left(E,O\right)=\min_{T}\left\{∥T∥:\left(E+T\right)\cap O\neq \emptyset \right\}.$$

The penetration depth is defined as

(20) $$\mathrm{pen}\left(E,O\right)=\min_{T}\left\{∥T∥:\left(E+T\right)\cap O=\emptyset \right\}.$$

Ultimately, the signed distance is defined as

(21) sd(E,O):=dist(E,O)−pen(E,O).

According to [39], collision detection can be extended:

(22) sd(E,Q)=sd(0,O−E).

To smooth the maximum and minimum operations, a widely adopted logsum-exp function is applied: if α>0 it approximates the maximum value in input vector, and if α<0 it selects the minimum value. For the e-th edge of the ego vehicle, calculate the minimum distance from all points on the obstacle to this edge:

(23) $$d_{Ee}\approx {\mathrm{lse}}_{\alpha <0}\left({\left[d_{e}^{1},...,d_{e}^{o},...,d_{e}^{n_{o}}\right]}^{T}\right).$$

Then, we select the maximum value from these distances to represent the signed distance from the obstacle to the ego vehicle:

(24) $$d_{E}^{O}\approx {\mathrm{lse}}_{\alpha >0}\left({\left[d_{E1},...,d_{Ee},...,d_{En_{e}}\right]}^{T}\right)$$

Similarly, the signed distance from the ego vehicle to the obstacles is defined as $d_{O}^{E}$. The signed distance between the ego vehicle and the obstacle is

(25) $$d\approx lse_{\alpha <0}\left({\left[d_{E}^{O},d_{O}^{E}\right]}^{T}\right)$$

Dynamic obstacle avoidance is achieved by penalizing signed distances smaller than the threshold.

## 4. Results

To validate the effectiveness of the proposed framework, we set up several challenging experiments, including navigating through areas with dense and small-sized obstacles, sparse and large-sized obstacles, and a scenario involving ego vehicle access to an urban expressway and arrival at a predetermined position. The design of these scenarios was intended to evaluate the effectiveness and robustness of our proposed framework within a multi-agent simulation platform. The simulation platform included diverse intelligent agents, static obstacles of varying sizes, and interactions among intelligent agents. All experiments were conducted on a desktop computer equipped with an Inter I5-12400 CPU, and the trajectory planning method could be run stably at 20 Hz.

### 4.1. Trajectory Planning for Scenes with Dense and Small-Sized Obstacles Ahead

In the experiments detailed in paper [14], the density and footprint of static obstacles were insufficient. To fully verify the effectiveness of the proposed framework, we proposed a scenario characterized by dense obstacles that covered a larger area than those presented in the referenced study. Our experimental parameters were set as follows: the maximum longitudinal acceleration was set to 8 m/s^2^. Given that the weight of the time optimization term $T_{a}$ in the optimization function significantly influences the conservativeness of the trajectory planning, the weight $w_{T}$ was set to a larger value of 500 in this validation scenario, to ensure that the acceleration could reach the road’s limit. We conducted trajectory planning for various road surface adhesion coefficients, specifically 0.4, 0.6, and 0.8, with a maximum vehicle speed of 20 m/s.

**Figure 7:**
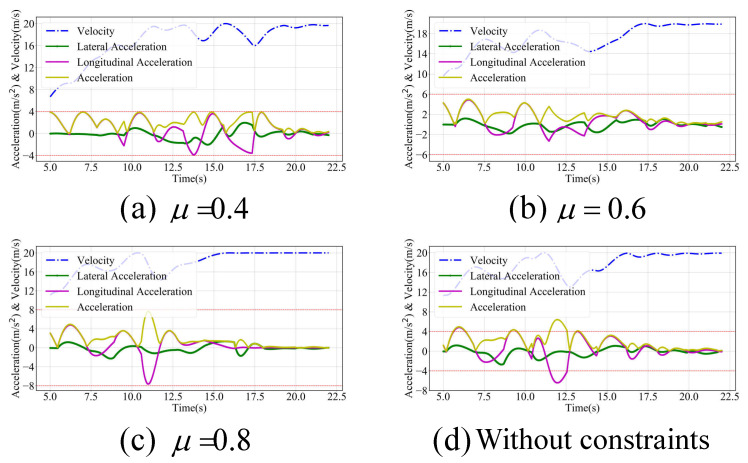
Results with varying road surface adhesion coefficients in a scenario with dense and small-sized obstacles ahead.

Figure 7 depicts the velocity, the longitudinal and lateral accelerations, and the total acceleration of the planned trajectory between 5 s and 22 s. The decision-making process and the planned trajectories varied under different road surface adhesion coefficients. As the longitudinal acceleration increased, the lateral acceleration decreased to meet the constraints imposed by the friction circle, suggesting that the vehicle might be in the acceleration phase. Conversely, a reduction in longitudinal acceleration associated with increased lateral acceleration might indicate a lane-changing phase. Throughout, the total acceleration of the planned trajectory did not exceed the limits of the friction circle.

**Figure 8:**
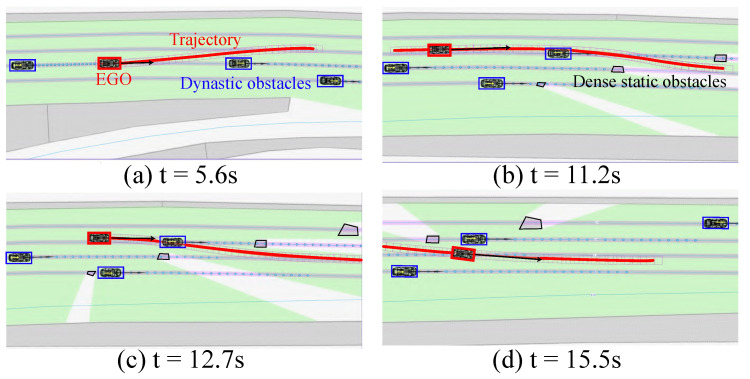
Snapshots in a scenario with dense and small-sized obstacles ahead.

The planned results for the road surface adhesion coefficient of 0.8 are illustrated in Figure 8. At t = 5.6 s, an overtaking trajectory was planned. At t = 11.2 s, the vehicle planned to change lanes to the right, but, due to a vehicle ahead, it decelerated to maintain a safe distance from the preceding vehicle. At t = 12.7 s, the acceleration trajectory was re-planned while ensuring no collision with static obstacles on both sides. At t = 15.5 s, the vehicle had successfully navigated through the complex section and subsequently maintained a straight trajectory at approximately 20 m/s. Throughout the process, the planned trajectory consistently ensured safety. It is important to note that we focused on the physical properties of the ego vehicle while ignoring those of other intelligent agents in the simulation.

**Figure 9:**
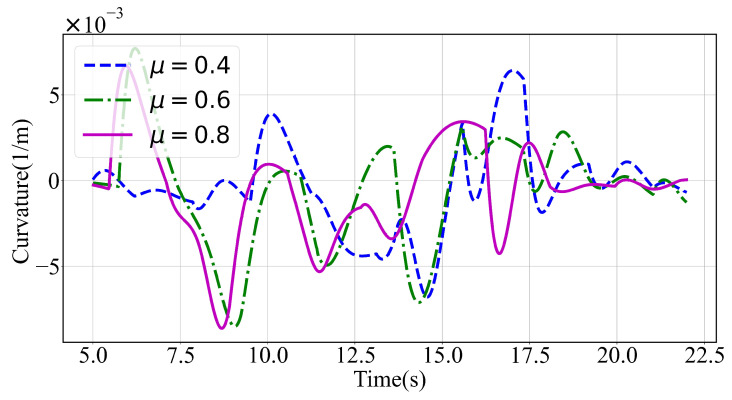
Curvature with varying road surface adhesion coefficients in a scenario with dense and small-sized obstacles ahead.

Trajectory curvature is one of the key indicators of trajectory smoothness, particularly in high-speed driving scenarios. Figure 9 shows that under different road surface adhesion coefficient conditions, the planned trajectory curvature showed slight differences and generally maintained a low value, indicating good trajectory smoothness.

### 4.2. Trajectory Planning for Scenes with Sparse and Large-Sized Obstacles Ahead

**Figure 10:**
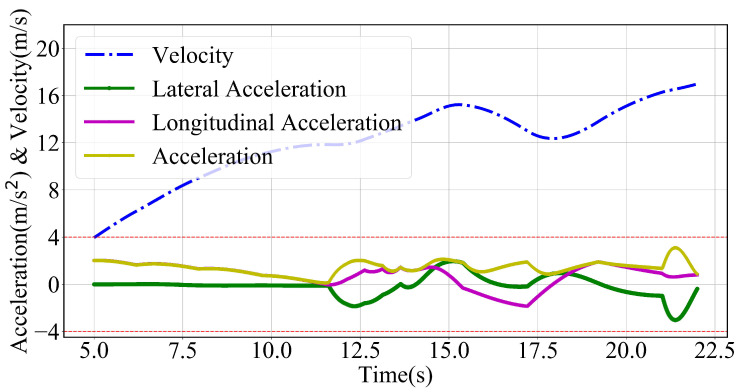
Results with a road surface adhesion coefficient of 0.8 in scenarios with sparse and large-sized obstacles ahead.

On structured roads, large infeasible areas may occur, such as the scenes of trailer accidents. Irregular polygons were used to simulate the large obstacles ahead. The experiment was conducted for various road surface adhesion coefficients: specifically 0.4, 0.6, and 0.8, with a maximum vehicle speed of 20 m/s. To further enhance safety, the weight $w_{T}$ was set to 10 and the maximum longitudinal acceleration was limited to 8 m/s^2^. Due to the low vehicle speeds, the results were almost identical under different road surface adhesion coefficients. The results with a road surface adhesion coefficient of 0.8 are depicted in Figure 10.

**Figure 11:**
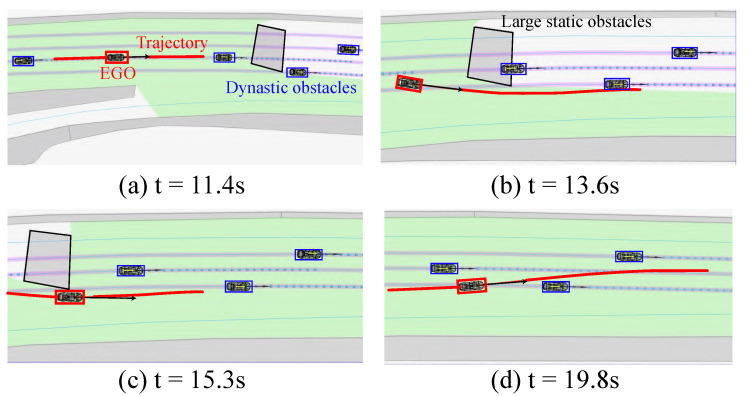
Snapshots in a scenario with sparse and large-sized obstacles ahead.

The planned results for a road surface adhesion coefficient of 0.8 are illustrated in Figure 11. At t = 11.4 s, the autonomous vehicle approached large obstacles and dynamic obstacles ahead. At t = 13.6 s, the vehicle followed a decelerating and obstacle-free trajectory. At t = 15.3 s, the vehicle almost passed static obstacles without collision. At t = 19.8 s, a leftward accelerating trajectory was planned, to overtake. The vehicle remained safe throughout the process.

**Figure 12:**
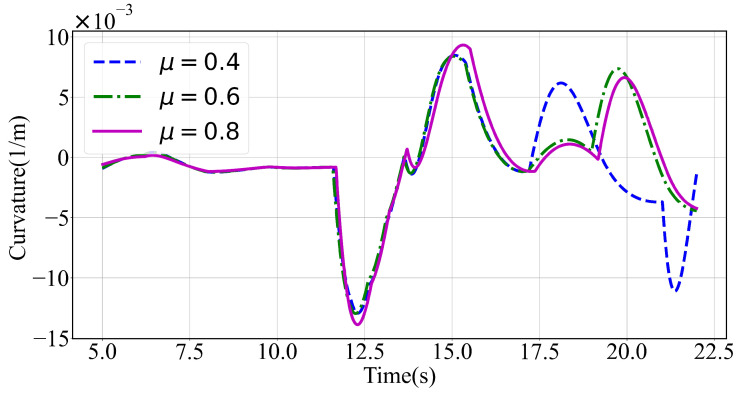
Curvature with varying road surface adhesion coefficients in a scenario with dense and small-sized obstacles ahead.

With regard to the curvature depicted in Figure 12, the simulation results show that under different road surface adhesion coefficient conditions the planned trajectory curvature was generally low. Additionally, when t < 17.5 s, the curvature of the planned trajectories under different road surface adhesion coefficients was roughly the same. At t = 17.5 s and μ=0.4, a left lane change trajectory was planned. In contrast, for the specific road surface adhesion coefficients, μ=0.6 and μ=0.8, the left lane change was planned around t = 19 s.

### 4.3. Efficiency of Vehicle Access to an Urban Expressway

**Figure 13:**
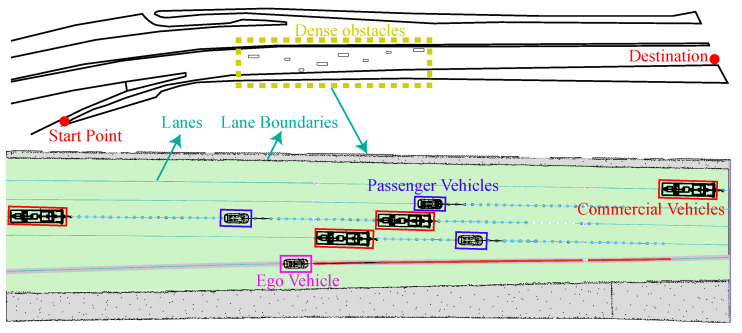
Urban expressway access scenario.

A comparative experiment with the SSC planner [35] was conducted in a scenario where the ego vehicle accessed an urban expressway and arrived at a predetermined position. As illustrated in Figure 13, this scenario encompassed a range of vehicles along a stretch of approximately 480 m. The maximum longitudinal acceleration was set at 3 m/s^2^, the weight $w_{T}$ was set to 10, and the maximum velocity was set 20 m/s. To ensure fairness, the same behavior planner was employed as in the experiment in [35].

**Figure 14:**
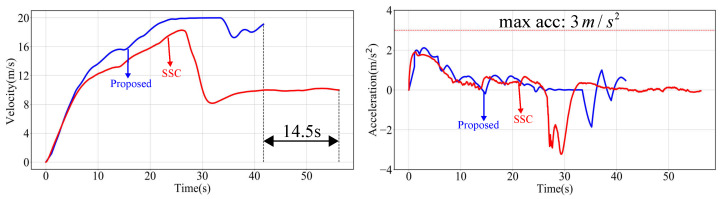
Speed and acceleration comparison profiles.

As shown in Figure 14, our travel time was reduced by 14.5 s compared to that of the SSC. Additionally, as detailed in Table 1, our average speed was approximately 4.74 m/s higher than that of the SSC. In the planned trajectory, our maximum lateral acceleration was slightly larger than that of the SSC, but it remained well below the limit of lateral acceleration. The SSC method, due to its conservative planning with Bézier curve parameterization, fails to meet the limits of planning constraints. Consequently, our method generates more efficient trajectories.

## 5. Conclusions

This study developed a methodology for complex urban scenarios with numerous dynamic and static obstacles. To address the potential risk of driving corridor generation failures caused by collisions between the initial trajectory obtained from the behavior planner and static obstacles, a topology-guided hybrid A* algorithm on an inflated map was employed, allowing for a driving corridor to be generated successfully. Furthermore, we integrated various constraints, including road adhesion coefficients, along with objectives such as efficiency and smoothness, by incorporating these constraints into the optimization objectives as soft constraints. This approach facilitated a coordinated optimization of both constraints and goals. Eventually, to fully leverage the XYT solution space and account for the coupling between the vehicle’s lateral and longitudinal motions, a space–time joint trajectory optimization method was utilized, to generate a smooth, safe, and efficient trajectory. The simulation results demonstrate that this methodology can generate trajectories with high efficiency and low collision probability, even in complex scenarios and under conditions of low road surface adhesion coefficients.

However, there were also shortcomings. Due to the absence of hard constraints in the optimization methodology, it was necessary to set reasonable parameters to ensure that all constraints were satisfied. Unreasonable parameters may lead to optimization failure, thereby posing a risk. Additionally, future studies should further validate the effectiveness of this methodology through real-world vehicle testing.

## Figures and Tables

**Table 1:** Statistics in simulation experiments.

Indicators	Ours		SSC	
	Max	Avg	Max	Avg
Speed (m/s)	19.99	16.53	18.28	11.79
Passing Time (s)	\	41.7	\	56.2
Curvature (1/m)	$1.3\times 10^{-3}$	$1.0\times 10^{-3}$	$2.3\times 10^{-3}$	$1.4\times 10^{-3}$
Longitudinal Acceleration (m/s^2^)	2.11	0.52	1.68	0.63
Lateral Acceleration (m/s^2^)	0.46	0.22	0.39	0.21

## Data Availability

Data is unavailable due to privacy and ethical restrictions.
